# Supplementary material for: Awareness of antimicrobial resistance and antibiotic use among poultry farmers in Accra, Ghana: A cross-sectional survey
Source: PLoS One. 2025 Dec 5;20(12):e0337531. doi: 10.1371/journal.pone.0337531 (PMC12680252; doi:10.1371/journal.pone.0337531)
Supplement: S1 File — (DOCX) [file pone.0337531.s001.docx]

**Questionnaire**

**Informed Consent**

This cross-sectional survey was aimed to assess the knowledge, attitudes, and practices regarding antimicrobial use and antimicrobial resistances among poultry farmers in Accra, Ghana.

Your honest responses will help in achieving the goals to enhance the rational use of antibiotics. Your name is not needed because the data will be treated as confidential.

**Written Informed Consent Section** - *Please read carefully*

I have decided to participate in this research on my own volition without any coercion.

**Signature:** ___________

Thank you for your willingness to participate in this study.

**Section A: Socio-demographic Characteristics**

**Instruction:** Circle or write the answer that corresponds with the best answer.

| S/N | Socio-demographic characteristics | Response |
| --- | --- | --- |
| 1 | Gender | A. Male |
|  |  | B. Female |
| 2 | Age | . |
| 3 | Education | 1. Illiterate |
|  |  | 1. Primary |
|  |  | 1. Secondary |
|  |  | 1. Tertiary |
| 4 | Geographical location | 1. Urban . |
|  |  | 1. Rural |

]

**Section B: Questionnaire on Knowledge of Antimicrobial usage and Antimicrobial Resistance in Poultry**

**Instruction:** Circle or write the answer that corresponds with the best answer.

| Awareness of Antimicrobial usage and AMR | **Category** |
| --- | --- |
| Knowledge of Antimicrobial Use | Yes |
|  | No |
|  | Total |
| Knowledge of the use of antimicrobials in animal production boosts the rate of AMR development. | Yes |
|  | No |
|  | I don’t know |
| Can you reduce AMR development by avoiding overuse of antimicrobials in animal production? | Yes |
|  | No |
|  | I don’t know |
| Can your imprudent use of antimicrobials affect the health of others in the form of AMR? | Yes |
|  | No |
|  | I don’t know |

**Section C:** **Questionnaire on Practice of Antimicrobial Usage & Antimicrobial Resistance in Poultry Production**

**Instruction:** Circle or write the answer that corresponds with the best answer

| **Practice of Antimicrobial Usage** | **Category** |
| --- | --- |
| What do you do when your animals get sick? | Self-treat |
|  | Take to Veterinary |
|  | Consult other farmers |
|  | Nothing |
| Who administers antimicrobials for your birds? | Self-administration |
|  | Veterinarian |
|  | Local traditional healer |
| Did you refer to guidelines while you administer antimicrobials for your animals? | Yes |
|  | No |
| Did you get prescription from veterinarians before you buy drugs? | Yes |
|  | No |
| For what purpose did you use antimicrobials most? | Treatment |
|  | Control (metaphyl) |
|  | Prevention (prophylaxis) |
|  | Increase in production |
| Source of antimicrobials for your animals? | Local Dispensers |
|  | Veterinary Clinic |
|  | Veterinary Pharmacy |

**Section D: Questionnaire on Attitude of Antimicrobial Usage & Antimicrobial Resistance in Poultry Production**

**Instruction:** Circle or write the answer that corresponds with the best answer

| **Questions / Responses** | **1** | **2** | **3** | **4** | **5** | **6** |
| --- | --- | --- | --- | --- | --- | --- |
| Is professional advice before using antimicrobials recommended? |  |  |  |  |  |  |
| Can imprudent AMU result irreversible loss of drugs effectiveness? |  |  |  |  |  |  |
| Can using antimicrobial alternatives like, good hygienic practice and vaccination reduce AMR development |  |  |  |  |  |  |
| Do you think using antimicrobials for the purpose of animal production is abusing antimicrobials? |  |  |  |  |  |  |
| Can AMU regulations be a solution for the irrational use of antimicrobials in animal production? |  |  |  |  |  |  |
| Can public awareness create a reduction in the development of AMR? |  |  |  |  |  |  |

*Key: 1= Strongly agree; 2= Agree; 3=Neutral; 4=Disagree; 5=Strongly disagree; 6= I don’t know.*

**Section E: Questionnaire on Commonly Used Antibiotics in Poultry Farming**

**Instruction:** Circle or write the answer that corresponds with the best answer

| Statement | Response |
| --- | --- |
| Please indicate which antibiotic you most frequently use in your poultry farming. | 1. Tetracycline |
|  | 1. Aminoglycosides |
|  | 1. Penicillins |
|  | 1. Fluoroquinolones |
|  | 1. Nitrofurans |
|  | 1. Co-trimothoxazole |
|  | 1. Colistin |
|  | 1. Macrolide |
