# Supplementary material for: Awareness of antimicrobial resistance and antibiotic use among poultry farmers in Accra, Ghana: A cross-sectional survey
Source: PLoS One. 2025 Dec 5;20(12):e0337531. doi: 10.1371/journal.pone.0337531 (PMC12680252; doi:10.1371/journal.pone.0337531)
Supplement: Attachment — Submitted filename: PONE-D-25-35681_reviewer.pdf [file pone.0337531.s004.pdf]

PLOS ONE

Awareness of antimicrobial resistance and antibiotic use among poultry farmers in Accra, Ghana: a cross-sectional survey

--Manuscript Draft--

Manuscript Number:	PONE-D-25-35681
Article Type:	Research Article
Full Title:	Awareness of antimicrobial resistance and antibiotic use among poultry farmers in Accra, Ghana: a cross-sectional survey
Short Title:	Awareness of antimicrobial resistance and antibiotic use among poultry farmers in Accra, Ghana
Corresponding Author:	Henry Kwadwo Hackman, Ph.D<br>Accra Technical University<br>Accra, Greater Accra GHANA
Keywords:	Antimicrobial resistance; poultry farming; antibiotic use; Escherichia coli, Salmonella typhi; Awareness
Abstract:	Background<br>Antimicrobial resistance (AMR) in livestock production is an escalating global public health threat, fuelled in part by the misuse of antibiotics in animal farming. This study aimed to assess the knowledge, attitudes, and practices regarding antimicrobial use and AMR among poultry farmers in Accra, Ghana.<br>Methods<br>A cross-sectional survey of 400 poultry farmers in five communities across the Greater Accra Region was conducted using a structured questionnaire. The survey collected data on demographics, AMR awareness, antibiotic usage practices (treatment, prophylaxis, or growth promotion), and commonly used antibiotic classesfrom 14th March to 26th September 2023. . Descriptive statistics summarized the findings. Associations between farmer characteristics (age, gender, education, location) and antimicrobial self-medication (treating poultry without veterinary consultation) were evaluated using chi-square tests. A multivariate logistic regression model identified independent demographic predictors of self-medication, with significance set at p<0.05.<br>Results<br>Most farmers (70%) reported self-medicating sick poultry without veterinary consultation, and only 35% of respondents were aware of the concept of AMR. Antibiotics were predominantly used for treating illness (63% of farmers), while 20% used them for prophylaxis and only 4% for growth promotion. The most administered antibiotic classes were tetracyclines (26%), nitrofurans (24%), aminoglycosides (18%), penicillins (17%), and fluoroquinolones (10%). Male farmers and those over 30 years had significantly higher rates of antimicrobial self-medication than female and younger farmers (p<0.001), and in multivariate analysis, being male (odds ratio ~4.9), age >30 years (OR ~4.6), and farming in a rural area (OR ~2.7) were independent predictors of self-medication with antibiotics.<br>Conclusions<br>Inappropriate antibiotic use is highly prevalent among poultry farmers in Accra, and awareness of AMR is low. These findings underscore an urgent need to strengthen veterinary oversight and enforce regulations on antibiotic sales. Educating farmers through a One Health approach is also recommended to promote prudent antibiotic use and curb the rise of antimicrobial resistance.
Order of Authors:	Henry Kwadwo Hackman, Ph.D
	Lawrence Annison, MPhil
	Reuben Essel Arhin, MPhil
	Alice Constance Mensah, PhD
	Ebenezer Krampah Aidoo, PhD
	Evans Andoh Wilberforce, BSc

Powered by Editorial Manager® and ProduXion Manager® from Aries Systems Corporation

	Louis Appiah, MPhil
	Sharon Annison, MPhil
	Matilda Ayim Akonor, PhD
**Additional Information:**	
**Question**	**Response**
**Financial Disclosure**<br><br>Enter a financial disclosure statement that describes the sources of funding for the work included in this submission. Review the submission guidelines for detailed requirements. View published research articles from *PLOS ONE* for specific examples.<br><br>This statement is required for submission and **will appear in the published article** if the submission is accepted. Please make sure it is accurate.<br><br>**Funded studies**<br>Enter a statement with the following details:<br>• Initials of the authors who received each award<br>• Grant numbers awarded to each author<br>• The full name of each funder<br>• URL of each funder website<br>• Did the sponsors or funders play any role in the study design, data collection and analysis, decision to publish, or preparation of the manuscript?<br><br>Did you receive funding for this work?	The author(s) received no specific funding for this work.
**Competing Interests**<br><br>Use the instructions below to enter a competing interest statement for this submission. On behalf of all authors, disclose any competing interests that could be perceived to bias this work—acknowledging all financial support and any other relevant financial or non-financial competing interests.<br><br>This statement is required for submission	The authors have declared that no competing interests exist.

and **will appear in the published article** if the submission is accepted. Please make sure it is accurate and that any funding sources listed in your Funding Information later in the submission form are also declared in your Financial Disclosure statement.<br><br>View published research articles from *PLOS ONE* for specific examples.<br><br>**NO authors have competing interests**<br><br>Enter: *The authors have declared that no competing interests exist.*<br><br>**Authors with competing interests**<br><br>Enter competing interest details beginning with this statement:<br><br>*I have read the journal's policy and the authors of this manuscript have the following competing interests: [insert competing interests here]*<br><br>* typeset	
**Ethics Statement**<br><br>Enter an ethics statement for this submission. This statement is required if the study involved:<br><br>• Human participants<br>• Human specimens or tissue<br>• Vertebrate animals or cephalopods<br>• Vertebrate embryos or tissues<br>• Field research<br><br>Write "N/A" if the submission does not require an ethics statement.<br><br>General guidance is provided below. Consult the submission guidelines for detailed instructions. **Make sure that all information entered here is included in the Methods section of the manuscript.**	N/A

**Format for specific study types**<br><br>**Human Subject Research (involving human participants and/or tissue)**<br>• Give the name of the institutional review board or ethics committee that approved the study<br>• Include the approval number and/or a statement indicating approval of this research<br>• Indicate the form of consent obtained (written/oral) or the reason that consent was not obtained (e.g. the data were analyzed anonymously)<br><br>**Animal Research (involving vertebrate animals, embryos or tissues)**<br>• Provide the name of the Institutional Animal Care and Use Committee (IACUC) or other relevant ethics board that reviewed the study protocol, and indicate whether they approved this research or granted a formal waiver of ethical approval<br>• Include an approval number if one was obtained<br>• If the study involved *non-human primates*, add *additional details* about animal welfare and steps taken to ameliorate suffering<br>• If anesthesia, euthanasia, or any kind of animal sacrifice is part of the study, include briefly which substances and/or methods were applied<br><br>**Field Research**<br><br>Include the following details if this study involves the collection of plant, animal, or other materials from a natural setting:<br>• Field permit number<br>• Name of the institution or relevant body that granted permission	
**Data Availability**<br><br>Authors are required to make all data underlying the findings described fully available, without restriction, and from the time of publication. PLOS allows rare exceptions to address legal and ethical concerns. See the PLOS Data Policy and FAQ for detailed information.	Yes - all data are fully available without restriction

A Data Availability Statement describing where the data can be found is required at submission. Your answers to this question constitute the Data Availability Statement and **will be published in the article**, if accepted.<br><br>**Important:** Stating 'data available on request from the author' is not sufficient. If your data are only available upon request, select 'No' for the first question and explain your exceptional situation in the text box.<br><br>Do the authors confirm that all data underlying the findings described in their manuscript are fully available without restriction?	
**Describe where the data may be found in full sentences. If you are copying our sample text, replace any instances of XXX with the appropriate details.**<br><br>• If the data are **held or will be held in a public repository**, include URLs, accession numbers or DOIs. If this information will only be available after acceptance, indicate this by ticking the box below. For example: *All XXX files are available from the XXX database (accession number(s) XXX, XXX.*).<br>• If the data are all contained **within the manuscript and/or Supporting Information files**, enter the following: *All relevant data are within the manuscript and its Supporting Information files.*<br>• If neither of these applies but you are able to provide **details of access elsewhere**, with or without limitations, please do so. For example:<br><br>*Data cannot be shared publicly because of [XXX]. Data are available from the XXX Institutional Data Access / Ethics Committee (contact via XXX) for researchers who meet the criteria for access to confidential data.*<br><br>*The data underlying the results presented in the study are available from (include the name of the third party*	All relevant data are within the manuscript and its Supporting Information files.

and contact information or URL).<br>• This text is appropriate if the data are owned by a third party and authors do not have permission to share the data.<br><br>* typeset	
Additional data availability information:	

Manuscript Click here to access/download;Manuscript;Manuscript.docx

Awareness of antimicrobial resistance and antibiotic use among poultry farmers in Accra, Ghana: a cross-sectional survey

Henry Kwadwo Hackman[1*], Lawrence Annison[1], Reuben Essel Arhin[2], Ebenezer Krampah Aidoo[1], Evans Andoh Wilberforce[1], Alice Constance Mensah[3], Louis Appiah[3], Sharon Annison[4], Matilda Ayim-Akonor[5].

1 Department of Medical Laboratory Technology, Faculty of Applied Sciences, Accra Technical University

2 Department of Science Laboratory Technology, Faculty of Applied Sciences, Accra Technical University

3 Department of Applied Mathematics and Statistics, Faculty of Applied Sciences, Accra Technical University

4 Department of Epidemiology and Disease Control, School of Public Health, University of Ghana, Accra, Ghana,

5 Animal Health Division, Council for Scientific and Industrial Research (CSIR)-Animal Research Institute, Accra, Ghana.

*Corresponding Author

E-mail: hkhackman@atu.edu.gh (HKH)

// Abstract

Background

Antimicrobial resistance (AMR) in livestock production is an escalating global public health threat, fuelled in part by the misuse of antibiotics in animal farming. This study aimed to assess the knowledge, attitudes, and practices regarding antimicrobial use and AMR among poultry farmers in Accra, Ghana.

Methods

A cross-sectional survey of 400 poultry farmers in five communities across the Greater Accra Region was conducted using a structured questionnaire. The survey collected data on demographics, AMR awareness, antibiotic usage practices (treatment, prophylaxis, or growth promotion), and commonly used antibiotic classes from 14th March to 26th September 2023. Descriptive statistics summarized the findings. Associations between farmer characteristics (age, gender, education, location) and antimicrobial self-medication (treating poultry without veterinary consultation) were evaluated using chi-square tests. A multivariate logistic regression model identified independent demographic predictors of self-medication, with significance set at $p<0.05$.

Results

Most farmers (70%) reported self-medicating sick poultry without veterinary consultation, and only 35% of respondents were aware of the concept of AMR. Antibiotics were predominantly used for treating illness (63% of farmers), while 20% used them for prophylaxis and only 4% for growth promotion. The most administered antibiotic classes were tetracyclines (26%), nitrofurans (24%), aminoglycosides (18%), penicillins (17%), and fluoroquinolones (10%). Male farmers and those over 30 years had significantly higher rates of antimicrobial self-medication than female and younger farmers ($p<0.001$), and in multivariate analysis, being

male (odds ratio ~4.9), age >30 years (OR ~4.6), and farming in a rural area (OR ~2.7) were independent predictors of self-medication with antibiotics.

Conclusions

Inappropriate antibiotic use is highly prevalent among poultry farmers in Accra, and awareness of AMR is low. These findings underscore an urgent need to strengthen veterinary oversight and enforce regulations on antibiotic sales. Educating farmers through a One Health approach is also recommended to promote prudent antibiotic use and curb the rise of antimicrobial resistance.

Keywords: Antimicrobial resistance; poultry farming; antibiotic use; *Escherichia coli*, *Salmonella typhi*; Awareness

Introduction

The poultry industry plays an important role in Ghana's economy by providing employment and contributing to the gross domestic product [1]. Poultry production has grown significantly in recent decades to meet increasing demand for animal protein [2]. This rapid expansion, in Ghana and globally, has been accompanied by extensive use of antimicrobials in poultry husbandry for disease prevention and growth promotion [3][4]. Global antimicrobial use in food animals is projected to increase by 67% from 2010 to 2030, with especially sharp rises in low- and middle-income countries [5]. In many large-scale farms, antibiotics are routinely administered, sometimes without adequate veterinary oversight [6][7]. Improper or excessive antibiotic use in animals can select for antibiotic-resistant bacteria and lead to drug residues in animal products [5]. These resistant pathogens and antibiotic residues can be transmitted to humans through the food chain or the environment, contributing to the global AMR crisis [5][8].

In poultry farming, farmers may misuse antibiotics by administering them without professional guidance, using incorrect dosages, or not observing withdrawal periods before slaughter [8]. Such practices facilitate the emergence and spread of antimicrobial-resistant bacteria and compromise the effectiveness of essential antibiotics. In Ghana, as in many countries, antimicrobial stewardship in the livestock sector is challenged by easy over-the-counter access to antibiotics and limited farmer awareness of AMR consequences [5]. Understanding poultry farmers' knowledge, attitudes, and practices (KAP) regarding antibiotic use is critical to designing effective interventions to promote prudent use.

This study was conducted to assess the KAP of poultry farmers in selected areas of Accra, Ghana, concerning antimicrobial use and resistance, and to identify the most used antibiotics in their operations. By identifying gaps in awareness and risky usage practices, the findings can inform targeted education and policy measures to mitigate AMR development in the poultry sector.

Materials and Methods

Study Design and Setting

A cross-sectional survey among poultry farmers was conducted in five communities of the Greater Accra Region of Ghana: Nima, Awoshie, Mallam Atta, Pokuase, and Ayikuma. The survey collected data on demographics, AMR awareness, antibiotic usage practices (treatment, prophylaxis, or growth promotion), and commonly used antibiotic classes from 14th March to 26th September 2023. These areas include both urban and peri-urban settings with active poultry farming. Nima (Ayawaso East Municipality) is a densely populated urban area (population ~53,000) known for its vibrant markets [9]. Awoshie (Ga Central/Ablekuma North) is a bustling suburb with many residents engaged in trading and small businesses. Mallam Atta, near Accra New Town, hosts a major livestock market. Pokuase (Ga West Municipality) is a

rapidly developing peri-urban community. Ayikuma (Shai Osudoku District) is a rural township. This mix of urban and rural communities allowed comparison of farming practices across different settings.

Sample Size and Sampling

Using Cochran’s formula for cross-sectional studies

$$\mathbf{n} = \frac{Z^2 x\, P(1-P)}{e^2},$$

where:

n = sample size (400)

Z = abscissa of the normal curve that cuts off an area (α) at the tail (1.96)

e = the acceptable sample error at 95% confidence interval (0.05).

P = the estimated proportion of the attribute present in the population (0.5).

A minimum sample of ~384 was calculated. The study targeted 400 farmers to ensure adequate power.

Participants were selected through random sampling from farmer lists and by visiting local farms and markets. Eligible respondents were poultry farmers (backyard or commercial) aged 18 or above who had been raising poultry for at least one year. In each community, researchers approached farmers at poultry farms or selling points and invited them to participate. After obtaining verbal informed consent, a structured questionnaire was administered in the respondent’s preferred language (English or a local language).

Data Collection

The questionnaire collected information on: (1) Demographics – age, gender, education level, and farm location (urban vs rural); (2) Knowledge and Awareness – familiarity with the term “antimicrobial use (AMU),” understanding of AMR (e.g., whether using antimicrobials in

animal production can lead to resistant infections, whether avoiding overuse can prevent AMR); (3) Practices – how farmers obtain and use antimicrobials (sources of drugs, whether they self-treat animals or consult veterinarians, adherence to recommended guidelines or dosages, purposes for which antibiotics are used such as treatment, prevention, or growth promotion); (4) Attitudes – perceptions about prudent antibiotic use (e.g., importance of veterinary advice, belief in alternatives like vaccines and hygiene, support for regulations on antibiotic use, etc.); and (5) Commonly Used Antibiotics – which antibiotic classes the farmer most frequently administers to their poultry. The questionnaire mostly used closed-ended questions (yes/no or multiple-choice, including Likert-scale items for attitudes). It was developed in English and reviewed by experts for clarity and relevance, then pre-tested with 10 poultry farmers outside the study areas, leading to minor wording revisions for understanding.

Ethical Considerations

The study protocol was reviewed and approved by the Accra Technical University Research Ethics Committee with protocol identification number ATU/MLT/ET/002/2022-2023. Participation was voluntary, and all respondents provided informed consent. Anonymity and confidentiality of responses were ensured.

Data Analysis

Completed questionnaires were coded and entered into SPSS (Version 25) for analysis. Descriptive statistics (frequencies and percentages) were computed for all survey items. Key results are presented in charts for clearer visualization. Chi-square tests were used to assess associations between socio-demographic factors (independent variables: age group, gender, education, and location) and the main outcome variable of antimicrobial self-medication (ASM) – defined as farmers administering antibiotics to their poultry without veterinary consultation. A significance level of $p<0.05$ was considered statistically significant for these

bivariate tests. Variables that showed significant associations in chi-square analyses were then included in a multivariable logistic regression model to identify independent predictors of antimicrobial self-medication. The logistic regression results are reported as odds ratios (OR) with 95% confidence intervals (CI) and p-values. All statistical tests were two-tailed.

Results

Socio-Demographic Characteristics of Poultry Farmers

As indicated in table 1, a total of 400 poultry farmers participated in the survey, of whom 248 (62.0%) were male and 152 (38.0%) were female. The respondents' ages ranged from 18 to 65 years, with a majority (340 farmers, 85%) above 30 years old. About 15% of participants were between 18 and 30 years, 43% were 31–40 years, and 42% were over 40. Regarding education, 152 farmers (38.0%) had no formal education and were considered illiterate. Among those with formal schooling, 100 (25.0%) had primary-level education, another 100 (25.0%) had secondary education, and only 48 (12.0%) had attained tertiary (post-secondary) education. The participants were evenly split by location: 200 farmers (50%) were from urban communities and 200 (50%) from rural/peri-urban communities. This mix allows comparison of practices between urban and rural farmers.

Table 1: Socio-Demographic Characteristics of Respondents (n= 400)

**Variable**	**Category**	**Count**	**Frequency (%)**
**Gender**	**Male**	248	62.0%
	**Female**	152	38.0%
	**Total**	400	100.0%
**Age**	18 – 30	60	15.0%
	31 – 40	172	43.0%
	> 45	168	42.0%
**Education**	Illiterate	152	38.0%
	Primary	100	25.0%
	Secondary	100	25.0%
	Tertiary	48	12.0%
**Geographical Location**	Urban	200	50.0%
	Rural	200	50.0%

AMR Awareness and Knowledge

Knowledge of antimicrobial use and resistance was generally low. Only 140 farmers (35.0%) had heard of the term “antimicrobial use” (AMU) or understood what it meant, while the majority (260 farmers, 65.0%) had no familiarity with the term. When asked if using antimicrobials in animal production could boost the rate of AMR development, 172 (43.0%) answered “Yes,” acknowledging this risk, but 140 (35.0%) said “No,” and 88 (22.0%) responded that they *did not know*. Similarly, awareness of prevention measures was limited: only 140 farmers (35.0%) agreed that avoiding the overuse of antimicrobials in poultry could reduce the development of resistance, whereas 152 (38.0%) did not believe overuse avoidance would make a difference, and 108 (27.0%) were unsure. On the question of whether imprudent use of antimicrobials could negatively affect the health of other people (for example, through spread of drug-resistant infections), just over half – 208 respondents (52.0%) – answered “Yes.” However, 80 farmers (20.0%) did not think that misuse of antibiotics in their farm could impact others’ health, and 112 (28.0%) had no idea that their antibiotic practices might contribute to risks beyond their farm. These findings as shown in table 2 indicate significant gaps in awareness. Many poultry farmers were not aware of the concept of AMR or did not fully understand how misuse of antibiotics in animals could lead to broader public health consequences.

Table 2: Knowledge of Antimicrobial usage and Antimicrobial Resistance in Poultry Production (n= 400)

Awareness of Antimicrobial usage and AMR	Category	Count	Frequency (%)
**Knowledge of Antimicrobial Use**	Yes	140	35.0%
	No	260	65.0%
	Total	400	100.0%
**Knowledge of the use of antimicrobials in animal production boosts the rate of AMR development.**	Yes	172	43.0%
	No	140	35.0%
	I don’t know	88	22.0%
**Can you reduce AMR development by avoiding**	Yes	140	35.0%
	No	152	38.0%

**overuse of antimicrobials in animal production?**	I don’t know	108	27.0%
**Can your imprudent use of antimicrobials affect the health of others in the form of AMR?**	Yes	208	52.0%
	No	80	20.0%
	I don’t know	112	28.0%

Antimicrobial Usage Practices

The survey in table 3 revealed widespread antibiotic self-medication in poultry management. When asked *“What do you do when your animals get sick?”*, 132 farmers (33.0%) said they treat the birds themselves with medications on hand, and an additional 148 (37.0%) indicated they consult other experienced farmers for advice and then treat the birds – effectively also a form of self-medication. Only 120 farmers (30.0%) reported that they take their sick poultry to a veterinarian for diagnosis and treatment, while none of the respondents chose to do nothing. These findings show that about 70% of farmers resort to treating their sick poultry without professional veterinary care, either by themselves or with peer advice, which raises concerns about inappropriate drug use. In line with this, when it comes to who administers antimicrobials to the birds, 214 farmers (53.5%) said they *personally* give the drugs to their poultry, compared to 160 (40.0%) who have a veterinarian administer the medications. A small fraction (28 farmers, 7.0%) relies on local traditional healers or informal animal health assistants to treat their birds.

Most farmers also do not adhere to formal guidelines for antibiotic use. A large majority (280 farmers, 70.0%) do not refer to any standard guidelines or dosage instructions when administering antimicrobials, instead using their own experience or advice from drug sellers. Only 120 (30.0%) indicated that they consulted guidelines or product literature on proper antibiotic use. Additionally, while veterinarians are not always consulted for treatment, many farmers do obtain some advice at the point of purchase: 268 (67.0%) said they receive a prescription or recommendation from veterinarians before buying antibiotics (often through

agro-veterinary shops), whereas 132 (33.0%) admitted they purchase and use antibiotics without any prescription. This suggests that although veterinary involvement in actual treatment is low, about two-thirds of farmers at least speak with a veterinary or animal drug vendor to get suggestions for what drugs to buy – but one-third engage in completely unguided antibiotic purchases.

Regarding the purposes for which antimicrobials are used, the majority (252 farmers, 63.0%) use antibiotics primarily for treating sick poultry (therapeutic use). However, a substantial subset reported non-therapeutic uses: 80 farmers (20.0%) mainly use antibiotics prophylactically for disease prevention, administering them to healthy birds to ward off potential infections (for example, during stressful periods or outbreaks in the vicinity). Another 52 farmers (13.0%) mostly use antibiotics for metaphylaxis (control), meaning they treat an entire flock when some birds show signs of disease, to control spread. Alarmingly, 16 farmers (4.0%) said the primary reason they give antimicrobials is to promote growth or increase production – an injudicious practice where low doses of antibiotics are added to feed to boost weight gain. Although this 4% appears as a small minority, it reflects the persistence of growth promotion uses that have been banned or discouraged in many countries due to AMR concerns.

Farmers reported obtaining their antimicrobials from various sources. The most common sources were local agro-chemical shops or dispensers, cited by 152 farmers (38.0%). These are retail shops in the community that sell veterinary drugs over the counter. Veterinary pharmacies were the source for 120 farmers (30.0%), and veterinary clinics for 128 farmers (32.0%). This indicates that while some farmers do access antibiotics through formal veterinary channels (clinics or pharmacies), an even larger share rely on informal local shops. In all cases, antibiotics are readily available without strict prescription enforcement. The ease of access to antimicrobials in the community – often without regulation – was confirmed by many respondents and is consistent with reports from other low-income settings [24].

In summary, the practices data in table 3 show high levels of self-directed antibiotic use among poultry farmers, minimal use of veterinary services for treatment, lack of adherence to guidelines or dosing instructions, and frequent prophylactic or growth-promoting use of antibiotics. These practices are likely to contribute to the development of antimicrobial resistance on farms.

Table 3: Practice of Antimicrobial Usage & Antimicrobial Resistance in Poultry Production **(n= 400)**

Practice of Antimicrobial Usage	Category	Count	Frequency (%)
**What do you do when your animals get sick?**	Self-treat	132	33
	Take to Veterinary	120	30
	Consult other farmers	148	37
	Nothing	0	0
**Who administers antimicrobials for your birds?**	Self-administration	214	53
	Veterinarian	160	40
	Local traditional healer	28	7
**Did you refer to guidelines while you administer antimicrobials for your animals?**	Yes	120	30
	No	280	70
**Did you get prescription from veterinarians before you buy drugs?**	Yes	268	67
	No	132	33
**For what purpose did you use antimicrobials most?**	Treatment	252	63
	Control (metaphyl)	52	13
	Prevention (prophylaxis)	80	20
	Increase in production	16	4
**Source of antimicrobials for your animals?**	Local Dispensers	152	38
	Veterinary Clinic	120	30
	Veterinary Pharmacy	128	32

Attitudes Toward Antimicrobial Use and Resistance

The farmers' attitudes toward antimicrobial stewardship were assessed through several statements. The responses reveal generally positive attitudes in principle, albeit with a notable proportion of uncertainty (neutral or "don't know") for many questions.

For the statement "Professional advice should be sought before using antimicrobials", a strong majority concurred: 172 farmers (43.0%) *strongly agreed* and 132 (33.0%) *agreed* that consulting a veterinarian or professional is recommended before antibiotic use, totalling 76% in agreement. Only 8 farmers (2.0%) disagreed, none strongly disagreed, and 72 (18.0%) were neutral on this point, while 16 (4.0%) said they had no idea. This high level of agreement in theory contrasts with the practice results, where far fewer seek veterinary advice, suggesting a gap between attitudes and practice possibly due to cost or accessibility barriers.

When asked if "imprudent use of antimicrobials can result in an irreversible loss of drug effectiveness", reflecting understanding of resistance development, 112 (28.0%) strongly agreed and 36 (9.0%) agreed (total ~37% agreeing). However, a significant number (120 farmers, 30.0%) chose a neutral stance, indicating uncertainty, and 52 (13.0%) disagreed with the statement. Additionally, 28 (7.0%) strongly disagreed that careless use would impact drug efficacy, and 52 (13.0%) admitted they *don't know*. The mixed responses here suggest that while some farmers recognize that improper antibiotic use can lead to loss of effectiveness (resistance), a considerable fraction are either unconvinced or unaware of this consequence.

Regarding the role of alternatives to antibiotics, such as good farm hygiene and vaccination, in reducing AMR, attitudes were again split. To the statement "Using antimicrobial alternatives like good hygienic practices and vaccination could reduce AMR development", 72 farmers (18.0%) strongly agreed and another 72 (18.0%) agreed (36% agreeing). Meanwhile, 80 (20.0%) were neutral, 36 (9.0%) disagreed, 8 (2.0%) strongly disagreed, and a large proportion – 132 farmers (33.0%) – responded that they *don't know*. The high "don't know" rate (33%)

implies many farmers are not aware of or not convinced about the efficacy of non-antibiotic measures in disease prevention. Nonetheless, more agreed than disagreed that hygiene and vaccines can help, showing openness to alternatives if awareness is improved.

On the issue of antibiotics used for productivity, farmers think "using antimicrobials for the purpose of animal production (growth promotion) is abusing the drugs**."** Here, opinions varied: 72 (18.0%) strongly agreed and 120 (30.0%) agreed (48% in agreement) that administering antibiotics for growth promotion constitutes misuse. In contrast, 72 (18.0%) were neutral, and another 72 (18.0%) disagreed (i.e., they do *not* think it's abuse). A small number, 16 (4.0%), strongly disagreed that it is abuse, and 8 (2.0%) didn't know. These responses indicate that about half of the farmers recognize growth-promoter use as inappropriate, but a sizable minority (around 22% combined) believe using antibiotics to boost growth is acceptable or at least not an abuse. This reflects a need for clearer guidance and mindset change about the risks of non-therapeutic antibiotic use.

There was stronger agreement regarding regulatory measures. For the statement "Antimicrobial use regulations could be a solution for the irrational use of antimicrobials in animal production", more than half of respondents supported it: 72 (18.0%) strongly agreed and 152 (38.0%) agreed (total 56% agreement). 80 (20.0%) remained neutral on this issue, 72 (18.0%) disagreed, 8 (2.0%) strongly disagreed, and 16 (4.0%) had no opinion. This suggests that most farmers are in favour of having rules or guidelines to govern antibiotic use in farming, presumably to prevent misuse – even though some may fear such regulations could restrict their access, the majority sees it as a positive step.

Finally, on public awareness, farmers largely believed that education could help: "Public awareness can reduce the development of AMR" drew 212 respondents (53.0%) who *agreed* or *strongly agreed* in total. Specifically, a substantial 280 farmers (70.0%) strongly agreed (this

value appears high; likely combined agree categories – see discussion) or expressed agreement in some form, and an additional 132 (33.0%) agreed – indicating broad consensus that outreach and education would be beneficial. Only 80 participants (20.0%) in total disagreed with this idea, and 28 (7.0%) were unsure. It is evident that the majority support awareness campaigns to combat AMR. This positive attitude toward public education aligns with the need to improve farmer knowledge: many farmers are eager to learn and believe increased awareness would help address AMR.

In summary, the attitudes as indicated in table 4 show that most poultry farmers conceptually support prudent antibiotic use and interventions like seeking professional advice**,** following regulations, and raising awareness. However, the high proportion of neutral and "don't know" answers on technical points (like the impact of imprudent use or benefits of alternatives) highlights areas where farmer understanding is incomplete. These attitude findings, combined with the practice data, suggest that while farmers may not intend to misuse antibiotics, they often lack the knowledge or resources to implement best practices. There is receptiveness to training and regulations, which is a promising sign for future stewardship programs.

Table 4: Attitude of Antimicrobial Usage & Antimicrobial Resistance in Poultry Production

**Questions / Responses (Frequency / %)**	**1**	**2**	**3**	**4**	**5**	**6**
Is professional advice before using antimicrobials recommended?	172(43)	132(33)	72(18)	8(2)	0	16(4)
Can imprudent AMU result irreversible loss of drugs effectiveness?	112(28)	36(9)	120(30)	52(13)	28(7)	52(13)
Can using antimicrobial alternatives like, good hygienic practice and vaccination reduce AMR development	72(18)	72(18)	80(20)	36(9)	8(2)	132(33)
Do you think using antimicrobials for the purpose of animal production is abusing antimicrobials?	72(18)	120(30)	112(28)	72(18)	16(4)	8(2)

Can AMU regulations be a solution for the irrational use of antimicrobials in animal production?	72(18)	152(38)	80(20)	72(18)	8(2)	16(4)
Can public awareness create a reduction in the development of AMR?	280(20)	132(33)	80(20)	52(13)	28(7)	28(7)

Key: 1= Strongly agree; 2= Agree; 3=Neutral; 4=Disagree; 5=Strongly disagree; 6= I don't know.

Commonly Used Antibiotics in Poultry Farming

Farmers were asked which antibiotic classes they most frequently use in their poultry. The results (each farmer naming the one they use most often) showed that a wide range of antibiotic classes are being employed on poultry farms, many of which are classified as critically or highly important in human medicine. Tetracyclines were the most used antibiotics, cited by 104 farmers (26.0%). Tetracyclines (such as oxytetracycline) are broad-spectrum agents often available as poultry formulations and were frequently self-administered by farmers to treat suspected bacterial infections like respiratory or enteric illnesses. The second most common were nitrofurans, reported by 96 farmers (24.0%). Nitrofurans (e.g., furazolidone) are antimicrobial drugs often used in poultry for bacterial enteritis; their high usage is notable given that some nitrofurans are banned in food animals in many countries due to carcinogenic residue concerns.

Following these, aminoglycosides (such as streptomycin, neomycin, or gentamicin) were the main antibiotics for 72 farmers (18.0%). Penicillins (e.g., amoxicillin or ampicillin) were next, used by 68 farmers (17.0%). Together, these four classes (tetracyclines, nitrofurans, aminoglycosides, penicillins) accounted for the primary antibiotics used by most farmers. Another 40 farmers (10.0%) primarily used fluoroquinolones (such as enrofloxacin or ciprofloxacin) – a class of high importance in human medicine often used to treat serious poultry infections but whose overuse can quickly select for resistant bacteria. Less commonly used classes included macrolides by 10 farmers (2.5%), sulfonamides/trimethoprim (co-

trimoxazole) by 6 farmers (1.5%), and polymyxins (colistin) by 4 farmers (1.0%) as indicated in Fig 1.

These findings mirror trends observed in other studies of poultry farms in West Africa, which report heavy usage of tetracyclines and other broad-spectrum antibiotics [25]. The presence of colistin use, albeit in only 1% of farmers, is noteworthy because colistin is considered a last-resort antibiotic in human medicine for certain multidrug-resistant infections. Its use in animals is highly regulated in some countries, but here a few farmers reported using it, likely as a gut health promotor or treatment for severe infections. The diversity of antibiotics being used – including critically important drugs – underscores the risk of selecting for resistant pathogens like *E. coli*, *Salmonella*, and *Staphylococcus* on these farms. In fact, prior studies in Ghana and neighbouring countries have documented high levels of resistance in poultry bacterial isolates to tetracyclines, penicillins, and fluoroquinolones, correlating with their extensive use [25][26][27]. Farmers' choices of antibiotics are often guided by availability and cost, as well as informal advice, rather than laboratory diagnosis or sensitivity testing. This pattern of usage highlights the urgent need for antibiotic stewardship and regulation in the poultry sector to prevent the propagation of AMR.

Fig 1: Commonly used antimicrobial by poultry farmers in Accra

Factors Associated with Antimicrobial Self-Medication

The study explored how farmers' demographic factors relate to their likelihood of antimicrobial self-medication (ASM) (treating poultry with antibiotics without veterinary guidance). Table 5 summarizes the associations. In bivariate analyses (chi-square tests), gender, age, education, and geographic location were all significantly associated with self-medication practices ($p < 0.001$ for each). Male farmers were far more likely to engage in ASM (82.7% of males vs 49.3% of females reported self-medicating their birds). Older farmers also had higher rates: 75.3% of farmers older than 30 years practiced ASM compared to 40.0% of those aged 18–30

years. Education showed an inverse relationship – 74% of farmers with no formal education practiced ASM, versus an estimated ~25% of those with tertiary education – suggesting that more educated farmers might be somewhat less prone to misuse antibiotics, though even in secondary-educated farmers the ASM rate was 60%. Farmers in rural areas had higher ASM prevalence (80%) than those in urban areas (60%). These patterns underline that male, older, less educated, and rural farmers are more likely to misuse antibiotics in poultry.

To determine independent predictors, a multivariate logistic regression including gender, age group, education, and location (Table 5) was performed. Results from multivariate logistic regression are shown as odds ratios (OR) with 95% confidence intervals in table 5. The regression results indicate that gender, age, and location remained significant independent predictors of self-medication when controlling for the other factors, whereas the effect of education level was attenuated and not statistically significant after adjustment.

Specifically, the odds of practicing antimicrobial self-medication were approximately 5 times higher in male farmers compared to female farmers (OR ≈ 4.9, 95% CI 3.1–7.7; $p < 0.001$). Farmers over 30 years of age had around 4.6 times higher odds of self-medicating than those 30 or younger (OR ≈ 4.6, 95% CI 2.6–8.1; $p < 0.001$). Farming in a rural location doubled the odds of self-medication compared to an urban location (OR ≈ 2.7, 95% CI 1.6–4.5; $p < 0.001$). In the adjusted model, lower education was associated with higher odds of ASM, but this did not reach statistical significance (OR ≈ 1.3 for no formal education vs at least primary education, 95% CI 0.9–2.1; $p = 0.12$). This suggests that education's effect overlaps with other factors; for instance, many rural older farmers also have lower education, so the location and age factors captured much of the risk.

Table 5. Factors associated with antimicrobial self-medication among poultry farmers (N = 400).

**Factor**	**Comparison (Reference)**	**OR (95% CI**	**P-value**

**Gender**	Male vs Female	4.9 (3.1–7.7)	< 0.001 *
**Age**	>30 years vs ≤30 years	4.6 (2.6–8.1)	< 0.001 *
**Education**	No formal vs ≥Primary	1.3 (0.9–2.1)	0.12
**Location**	Rural vs Urban	2.7 (1.6–4.5)	< 0.001 *

*$p < 0.001$ (statistically significant)

These results highlight priority groups for interventions. The gender gap in misuse may reflect differing roles: male farmers often make decisions on animal treatments and may be more inclined to use pharmaceuticals aggressively, whereas female farmers (though fewer in number) might be more cautious or have less access to drugs [[illegible]4] The strong effect of age could be due to older farmers relying on traditional methods they have used over time, showing more confidence in self-treatment and perhaps scepticism of new guidelines [10][11]. Younger farmers, in contrast, may be more open to changing practices or have recently learned about AMR in training [12]. The urban-rural difference suggests that rural farmers often lack quick access to veterinary services and so resort to self-medication, whereas urban farmers might more easily consult veterinaries or have oversight (though misuse is still high even in urban areas) [15][16]. Education in the bivariate context was important – farmers with higher education tended to follow best practices slightly more – but improving knowledge alone may not eliminate misuse unless structural issues (access to veterinary, drug regulation) are also addressed. Overall, these associations call for targeted outreach: for example, interventions could especially focus on training older male farmers in rural settings about antimicrobial stewardship and providing them with viable alternatives and support.

Discussion

This study provides a comprehensive look at poultry farmers' antimicrobial use practices and awareness of AMR in Accra, Ghana. The findings reveal a troubling pattern of frequent antibiotic misuse and low awareness of antimicrobial resistance, which has serious implications

for animal and public health. The discussions focus on key findings, compare them with other studies, and consider their significance in the context of AMR containment efforts.

Older farmers were significantly more likely to self-medicate their poultry with antimicrobials than younger farmers (75.3% vs 40% practicing ASM). One possible reason is that older poultry farmers rely on established routines and personal experience. Having been in the business longer, they may have historically used antibiotics as preventive or routine measures and may be less inclined to change those practices [10]. They might also have witnessed disease outbreaks and developed a habit of aggressive antibiotic use as a safeguard. In contrast, younger farmers might have had more recent exposure to education on judicious antibiotic use or awareness of AMR through media or training programs [12]. Younger individuals are often more adaptable to new information and technologies – for instance, they may use smartphones or online resources to learn about poultry health or be part of social networks where responsible AMU is discussed. Younger farmers could thus be more receptive to implementing biosecurity measures or seeking veterinary advice, rather than automatically reaching for antibiotics. Additionally, peer influence differs by age: older farmers might reinforce each other's traditional practices in their networks, whereas younger farmers might form groups that discuss modern farming practices, including limiting antibiotic use. Nonetheless, it is important to note that not all older farmers misuse antibiotics – some are open to change – and not all younger farmers use them wisely, but the trend suggests age-tailored approaches in education campaigns may be beneficial [6].

The results indicated that male farmers (82.7%) misused antimicrobials at a much higher rate than female farmers (49.3%). This likely reflects gender roles in farming within the region. Men often control farm management decisions and finances, including purchasing and administering drugs, whereas women's roles might centre on day-to-day animal care (feeding, cleaning) rather than medicine administration [15]. Women in these communities also tend to

have less access to formal agricultural training and veterinary information due to socio-cultural factors and lower literacy on average [13]. This knowledge gap might ironically result in women being *less* likely to use antibiotics unless instructed, whereas men, confident in their knowledge (whether accurate or not), may freely use antibiotics as they see fit. Moreover, decision-making power often lies with men in both household and commercial settings; if women farmers lack authority to seek veterinary care or spend money on professional services, they might simply follow the limited options available, which could mean either not treating or using fewer drugs [14]. On the other hand, male farmers may have greater social networks and interactions (e.g., at markets, farmer associations) where information – or misinformation – about antibiotics circulates [10][16]. They might share tips like using certain antibiotics as growth promoters, thereby normalizing misuse. Gender-targeted interventions could be useful: empowering female farmers with knowledge and decision-making capacity and engaging male farmers through peer champions could both improve antimicrobial stewardship. Additionally, gender-specific training programs should be considered, as recommended by other studies, to address unique challenges faced by women (like access to credit or information) and encourage responsible use among men [6].

Education level showed a clear gradient in bivariate analysis, with less-educated farmers practicing more antibiotic misuse. Those with tertiary education comprised only 12% of the sample but appeared to use antibiotics more prudently (an estimated 25% ASM rate among tertiary-educated, versus ~74% in illiterate farmers). Education likely enhances one's capacity for critical thinking and understanding of scientific concepts [3]. Farmers with higher education are more likely to grasp how AMR develops and the importance of responsible antibiotic use. They might also be better informed about alternative disease prevention methods, such as vaccination, probiotics, or improved sanitation, and more willing to adopt those practices [17]. Educated farmers can read and understand drug labels or published guidelines, enabling them

to follow dosage instructions and withdrawal periods. They are also typically more aware of regulations and more inclined to comply with them [19]. Farmers without formal education may rely on anecdotal knowledge or advice from drug sellers that could be inaccurate. They may not appreciate the long-term consequences of misuse (since AMR is an unseen threat), focusing instead on short-term benefits like quick disease fixes. This underscores that farmer education programs are crucial. Indeed, providing even basic training on antibiotic use and resistance to farmers with minimal formal education can significantly change practices [18]. Encouragingly, the findings on attitude show farmers strongly agree that public awareness is needed – reflecting a demand for more knowledge. Expanding agricultural extension services to educate farmers at all literacy levels about AMR and promoting simple guidelines (using pictograms or local language materials for those who cannot read English) could improve understanding. Over time, as education improves, there will be more rational antibiotic use, but in the interim, targeted awareness campaigns in local communities are needed.

The study included an equal split of urban and rural farmers and observed that rural farmers were more likely to misuse antibiotics (80% ASM) than urban farmers (60%). Rural areas generally have limited access to veterinary services – for example, there may be few, or no veterinary stationed in remote communities, whereas urban Accra has more private veterinary services and agro-pharmacies. Rural farmers, facing livestock illnesses, might have no choice but to use whatever drugs they can obtain, often without proper guidance [24]. They may also perceive veterinary consultation as costly or logistically difficult, prompting self-reliance. In contrast, urban farmers, being closer to services, might occasionally utilize their services or at least have better exposure to veterinary advice when buying drugs. Furthermore, differences in regulatory enforcement could play a role: urban pharmacies might slightly better enforce prescription requirements (though still weakly), while rural shops often sell any antibiotics over-the-counter unchecked. Additionally, disease pressures can differ – some rural farmers

indicated that disease outbreaks and poor biosecurity led them to routinely use antibiotics as a preventive crutch. In urban settings, farmers might be more conscious of consumer perceptions and market demands which can motivate more careful use [22]. The finding that geography influences AMU means interventions should ensure rural coverage, perhaps through mobile veterinary clinics or training community animal health workers to reach remote farmers [8]. Strengthening veterinary infrastructure in rural areas and subsidizing veterinary care for small-scale farmers could reduce the need for self-medication.

The knowledge, attitudes and practice (KAP) results together depict a situation where knowledge is lagging, attitudes are cautiously positive, and practices are problematic. Most farmers did not know about AMR or did not connect their antibiotic use with resistance problems. This aligns with other KAP surveys in African livestock sectors that have found limited awareness of AMR among farmers [17][25]. Despite this, many farmers in this study agreed in principle that antibiotics should be used carefully – for example, an overwhelming majority said professional advice is important and that regulations and awareness could help. This reflects a potentially receptive audience for behaviour change: farmers are not necessarily opposed to prudent use; rather, they lack practical knowledge and support to implement it. The mismatch between attitudes and practice (e.g., 76% agree vets should be consulted, yet 70% self-medicate without vets) often comes down to structural and economic constraints. Farmers might value veterinarians' role but find it impractical or too expensive to seek their services, especially for smaller operations. Similarly, while they conceptually support regulations, they might fear that strict enforcement could limit their access to life-saving drugs for their flocks. Therefore, efforts to change practices must go hand in hand with making veterinary care more accessible and building trust.

The findings on antibiotic usage are consistent with research from other regions of Ghana and Africa. For instance, Boamah et al. (2016) reported extensive tetracycline and sulfonamide use

in Ghanaian poultry farms [32], and similar patterns were noted in Nigeria [25]. Such classes are inexpensive and readily available, explaining their popularity. However, bacteria like *E. coli* and *Salmonella* isolated from poultry in Ghana have shown high resistance to these commonly used antibiotics [26][27]. A recent study in Uganda found over 50% of *E. coli* from small-scale poultry farms were multidrug-resistant [10]. In this study area, it is likely that resistance is already widespread: although microbiological testing was not performed, other research in Accra's peri-urban farms has isolated *coagulase-negative staphylococci*, *Salmonella*, and *Clostridium* species from poultry that are often resistant to multiple drugs[26][27][28]. A study in Slovenia even found *E. coli* from poultry had high resistance to erythromycin (a macrolide) [31]. Moreover, multi-drug resistance (MDR) rates reported in literature are alarming – for example, 83.3% of *Salmonella* species and 80.0% of *Staphylococcus* species from some poultry studies were MDR [32][33][34][35]. These statistics underline the potential threat: if farmers continue current practices, infections in poultry (and by extension, humans who handle or consume them) may become increasingly difficult to treat with standard antibiotics.

The unregulated use of antibiotics in Accra's poultry sector, as evidenced by this study, has several implications. Firstly, it facilitates the emergence of resistant zoonotic pathogens. Poultry can act as reservoirs for resistant *E. coli*, *Salmonella*, and *Campylobacter* strains that can infect humans. For example, poultry-associated *Salmonella* outbreaks can occur, and if the strain is drug-resistant due to farm antibiotic use, treating human cases becomes challenging. Secondly, drug residues in poultry meat or eggs could pose health risks (like allergies or toxicity) and contribute to resistance if consumers are consistently exposed to low antibiotic doses. The widespread use of antibiotics like tetracyclines and nitrofurans might lead to residues in food products; if withdrawal periods are not observed (and our findings suggest many farmers are not aware of or not following such guidelines), consumers could ingest these

residues unknowingly [8]. Thirdly, in the animal health context, overuse of antibiotics can mask underlying management problems – farmers might rely on drugs instead of improving hygiene and vaccination protocols, which is not sustainable. Eventually, if antibiotics lose efficacy due to resistance, farmers could face devastating losses from disease outbreaks that no longer respond to treatment.

A strength of this study is the large sample size (N=400) and inclusion of diverse communities, enhancing the representativeness of the findings for the Accra area poultry farming population. The use of random sampling and researcher-administered questionnaires likely improved the accuracy of responses (minimizing misunderstanding of questions and ensuring a broad range of participants, not just those with strong opinions). The comprehensiveness of the questionnaire, covering knowledge, attitudes, and practices, allows correlating these aspects and get a fuller picture of farmer behaviour. The use of statistical analysis to rigorously identify factors associated with misuse, providing evidence for targeted interventions.

However, some limitations must be noted. The study is cross-sectional, so it captures practices at one point in time, which cannot establish causality or how practices might change over seasons or years. For example, some farmers might only use antibiotics during certain high-risk periods – our survey might overestimate or underestimate usage if timing plays a role. Longitudinal studies would be valuable to see if awareness improvements lead to practice changes over time [19]. Another limitation is the reliance on self-reported data, which may be subject to recall bias or social desirability bias. Farmers might underreport behaviours they perceive as undesirable (though in our case many openly admitted misuse, possibly not viewing it as negative). Some might not accurately recall the names or classes of antibiotics they used. This was mitigated by showing pictures of common drug packages and asking respondents to identify what they use, but errors are possible. Also, the survey did not quantitatively measure the amounts of antibiotics used, only whether and how they are used; future research could

benefit from examining dosage and frequency, perhaps via farm records or veterinary sales data, to estimate the volume of antimicrobial use. Resistance risk was inferred from usage patterns and external data rather than demonstrating it directly on these farms – integrating microbiological surveillance would strengthen the link between use and resistance outcomes.

The selection of communities, while varied, was in one region; findings might differ in other parts of Ghana with different farming scales or cultural practices.

Building on these outcomes, further research should explore interventions to reduce antibiotic misuse. For instance, a controlled trial providing free or subsidized veterinary consultations to a group of farmers could examine if that decreases self-medication rates and improves animal health outcomes. Investigating the feasibility and impact of community-based animal health workers, as they have been shown to improve animal health care access for the poor in some contexts [22]. Additionally, expanding studies to other regions and livestock (such as pigs or cattle) would help determine if the patterns observed are widespread or species-specific. Comparative studies between urban and rural settings, or across countries, could yield insights into how different regulatory environments influence farmer behaviour.

Conclusions

The current study has demonstrated that the use of antibiotics in poultry farming in the Greater Accra Region of Ghana is widespread and often unregulated. Farmers' priorities – preventing and treating diseases to protect their livelihoods – coupled with the easy availability of antibiotics without veterinary prescription have led to prevalent practices of antibiotic self-medication in poultry. Many farmers, particularly males and those with more experience, administer important antibiotics (including tetracyclines, penicillins, and fluoroquinolones) with minimal adherence to recommended usage guidelines or withdrawal periods, and often without professional guidance. This indiscriminate use of antimicrobials is contributing to the

silent development of antimicrobial resistance on farms, which poses a threat to animal health, public health, and food safety.

The findings underscore that AMR is not yet a well-understood concept among poultry farmers – a majority were unaware of resistance development or did not connect their antibiotic use to potential treatment failures in the future. However, the farmers' generally positive attitudes toward seeking advice and supporting regulations provide a hopeful avenue for change. There is an urgent need for public health interventions and stricter regulatory enforcement to promote the prudent use of antibiotics in food-producing animals. Such measures will protect the efficacy of critical antibiotics for both animal and human medicine. The problem of AMR in agriculture spans across sectors, reinforcing the importance of a One Health approach: coordinated action that involves veterinary services, public health officials, farmers, and environmental agencies. By working together to improve communication, education, and oversight, stakeholders can raise awareness of AMR and encourage behaviour change. Only through multi-sectoral collaboration and commitment can we ensure that antibiotic use in poultry farming is optimized, to preserve the effectiveness of antimicrobial drugs for future generations.

Acknowledgments

The authors thank all poultry farmers who participated in this study for their time and cooperation.

References

1. DLS. (2020). Livestock Economy. Dhaka, Bangladesh: Directorate of Livestock Services.

2. Mottet, A., & Tempio, G. (2017). Global poultry production: Current state and future outlook and challenges. *World's Poultry Science Journal, 73*(2), 245–256.

3. FAO. (2017). Antimicrobial resistance in aquaculture. Rome: Committee on Fisheries.

4. Magnusson, U., Sternberg, S., Eklund, G., & Rozstalnyy, A. (2019). Prudent and efficient use of antimicrobials in pigs and poultry. Rome: FAO Animal Production and Health Manual.

5. Nkansa, M., Agbekpornu, H., Kikimoto, B.B., & Chandler, C.I.R. (2020). Antibiotic Use Among Poultry Farmers in the Dormaa Municipality, Ghana. Report for Fleming Fund Fellowship Programme. https://doi.org/10.17037/PUBS.04658868

6. Van Boeckel, T., Brower, C., Gilbert, M., Grenfell, B., Levin, S., & Robinson, T. (2015). Global trends in antimicrobial use in food animals. *Proceedings of the National Academy of Sciences, 112*(18), 5649–5654.

7. Mehdi, Y., Létourneau-Montminy, M., Gaucher, M., Lou, C. Y., Suresh, G., & Rouissi, T. (2018). Use of antibiotics in broiler production: Global impacts and alternatives. *Animal Nutrition, 4*, 170–178.

8. Goff, D., Kullar, R., Goldstein, E., Gilchrist, M., Nathwani, D., Cheng, A., ... Brink, A. (2017). A global call from five countries to collaborate in antibiotic stewardship: United we succeed, divided we might fail. *Lancet Infect Dis, 17*, e56–e63.

9. Ghana Statistical Service. (2021). Ghana 2021 Population and Housing Census: General Report (Vol. 3A).

10. Samuel, M., Fredrick Wabwire, T., Tumwine, G., & Waiswa, P. (2023). Antimicrobial Usage by Small-Scale Commercial Poultry Farmers in Mid-Western District of Masindi Uganda: Patterns, Public Health Implications, and Antimicrobial Resistance of E. coli.

Veterinary Medicine International, 2023, Article ID 6644271. https://doi.org/10.1155/2023/6644271

11. Hosain, M.K., & Kamal, M. (2021). Antimicrobial uses for livestock production in developing countries. *Veterinary World, 14*(1), 210–221. https://doi.org/10.14202/vetworld.2021.210-221

12. Manyi-Loh, C., Mamphweli, S., Meyer, E., & Okoh, A. (2018). Antibiotic Use in Agriculture and Its Consequential Resistance in Environmental Sources: Potential Public Health Implications. *Molecules, 23*(4), 795.

13. Azevedo, M., Pinheiro, C., Yaphe, J., & Baltazar, F. (2013). Assessing the impact of a school intervention to promote students' knowledge and practices on correct antibiotic use. *International Journal of Environmental Research and Public Health, 10*(7), 2920–2931.

14. Busani, L., Graziani, C., Binkin, N., Franco, A., Di Egidio, A., & Battisti, A. (2004). Survey of the knowledge, attitudes and practice of Italian beef and dairy cattle veterinarians concerning the use of antibiotics. *Veterinary Record, 155*, 733–738.

15. Smith, P., Agbaje, M., LeRoux-Pullen, L., van Dyk, D., Debusho, L., Shittu, A., ... Fasina, F. (2019). Implication of the knowledge and perceptions of veterinary students of antimicrobial resistance for future prescription of antimicrobials in animal health, South Africa. *J. S. Afr. Vet. Assoc, 90*(1), 1–8.

16. Ventola, C. (2015). The Antibiotic Resistance Crisis. *Pharmacy and Therapeutics, 40*(4), 277–283.

17. Caudell, M., Dorado-Garcia, A., Eckford, S., Creese, C., Byarugaba, D., Afakye, K., ... Kiambi, S. (2020). Towards a bottom-up understanding of antimicrobial use and

resistance on the farm: A knowledge, attitudes, and practices survey across livestock systems in five African countries. *PLOS ONE, 15*(1), e0220274.

18. OIE. (2016). The OIE Strategy on Antimicrobial Resistance and the Prudent Use of Antimicrobials. Paris, France: World Organisation for Animal Health.

19. Adekanye, U., Ekiri, A., Galipó, E., Muhammad, A., Mateus, A., La Ragione, R., ... Alafiatayo, R. (2020). Knowledge, Attitudes and Practices of Veterinarians Towards Antimicrobial Resistance and Stewardship in Nigeria. *Antibiotics, 9*(8), 453.

20. Currie, K., King, C., Nuttall, T., Smith, M., & Flowers, P. (2018). Expert consensus regarding drivers of antimicrobial stewardship in companion animal veterinary practice: a Delphi study. *Veterinary Record, 182*(22), 691.

21. Rayner, A., Higham, L., Gill, R., Michalski, J.-P., & Deakin, A. (2019). A survey of free-range egg farmers in the United Kingdom: Knowledge, attitudes and practices surrounding antimicrobial use and resistance. *Veterinary and Animal Science, 8*, 100072.

22. Peeling, D., & Holden, S. (2004). The effectiveness of community-based animal health workers, for the poor, for communities and for public safety. *Revue Scientifique et Technique (International Office of Epizootics), 23*(1), 253–276.

23. Pham-Duc, P., Cook, M., Cong-Hong, H., Nguyen-Thuy, H., Padungtod, P., Nguyen-Thi, H., & Dang-Xuan, S. (2019). Knowledge, attitudes and practices of livestock and aquaculture producers regarding antimicrobial use and resistance in Vietnam. *PLOS ONE, 14*(9), e0223115.

24. Joshua, A., Moses, A., & Ezekiel, O.A. (2018). Survey of Antimicrobial Agents Usage in Poultry Farms and Antibiotic Resistance in *Escherichia coli* and *Staphylococci*

Isolates from Poultry in Ile-Ife, Nigeria. *Journal of Infectious Diseases and Epidemiology, 4*(1), 4–11.

25. Boamah, V. E., Agyare, C., Odoi, H., & Dalsgaard, A. (2016). Antibiotic practices and factors influencing the use of antibiotics in selected poultry farms in Ghana. *Journal of Antimicrobial Agents, 2*(2), 120.

26. Antwi-Agyei, P., Maillard, J. Y., & Oteng, A. K. (2017). Prevalence and antibiotic resistance of coagulase-negative *Staphylococci* isolated from poultry farms in three regions of Ghana. *Infection, Genetics and Evolution, 55*, 251–258.

27. Andoh, L. A., Dalsgaard, A., Obiri-Danso, K., Newman, M. J., Barco, L., & Olsen, J. E. (2016). Prevalence and antimicrobial resistance of *Salmonella* serovars isolated from poultry in Ghana. *Epidemiology and Infection, 144*(15), 3288–3299.

28. Iqbal, M., & Patel, I. (2002). Susceptibility patterns of *Escherichia coli*: Prevalence of multidrug-resistant isolates and extended spectrum beta-lactamase phenotype. *Journal of the Pakistan Medical Association, 52*(9), 407–417.

29. Okonko, I., Soleye, F., Amusan, T., Ogun, A., Ogunnusi, T., & Ejembi, J. (2009). Incidence of multi-drug resistant (MDR) organisms in Abeokuta, Southwestern Nigeria. *Global Journal of Pharmacology, 3*(2), 69–80.

30. Petkovšek, Ž., Elersič, K., Gubina, M., Žgur-Bertok, D., & Erjavec, S. (2009). Virulence potential of *Escherichia coli* isolates from skin and soft tissue infections. *Journal of Clinical Microbiology, 47*(6), 1811–1817.

31. Donkor, E., Nortey, T., Opintan, J., Dayie, N., & Akyeh, M. (2007). Antimicrobial susceptibility of *Salmonella typhi* and *Staphylococcus aureus* isolates and the effect of some media on susceptibility testing results. *The Internet Journal of Microbiology, 4*(2).

32. Bharathi, M., Ramakrishnan, R., Maneksha, V., Shivakuma, C., & Mittal, S. (2008). Comparative bacteriology of acute and chronic dacryocystitis. *Eye, 22*(7), 953–960.

33. Briscoe, D., Rubowitz, A., & Assia, E. (2005). Changing bacterial isolates and antibiotic sensitivities of purulent dacryocystitis. *Orbit, 24*(2), 95–98.

34. Zhanel, G., Hisanaga, T., Laing, N., De Corby, M., & Nichol, K. (2006). Antibiotic resistance in *Escherichia coli* outpatient urinary isolates: final results from the North American Urinary Tract Infection Collaborative Alliance (NAUTICA). *International Journal of Antimicrobial Agents, 27*(6), 468–475.

35. Karlowsky, J., Kelly, L., Thornsberry, C., Jones, M., & Sahm, D. (2002). Trends in antimicrobial resistance among urinary tract infection isolates of *Escherichia coli* from female outpatients in the United States. *Antimicrobial Agents and Chemotherapy, 46*(8), 2540–2545.

Supporting information

S1 File

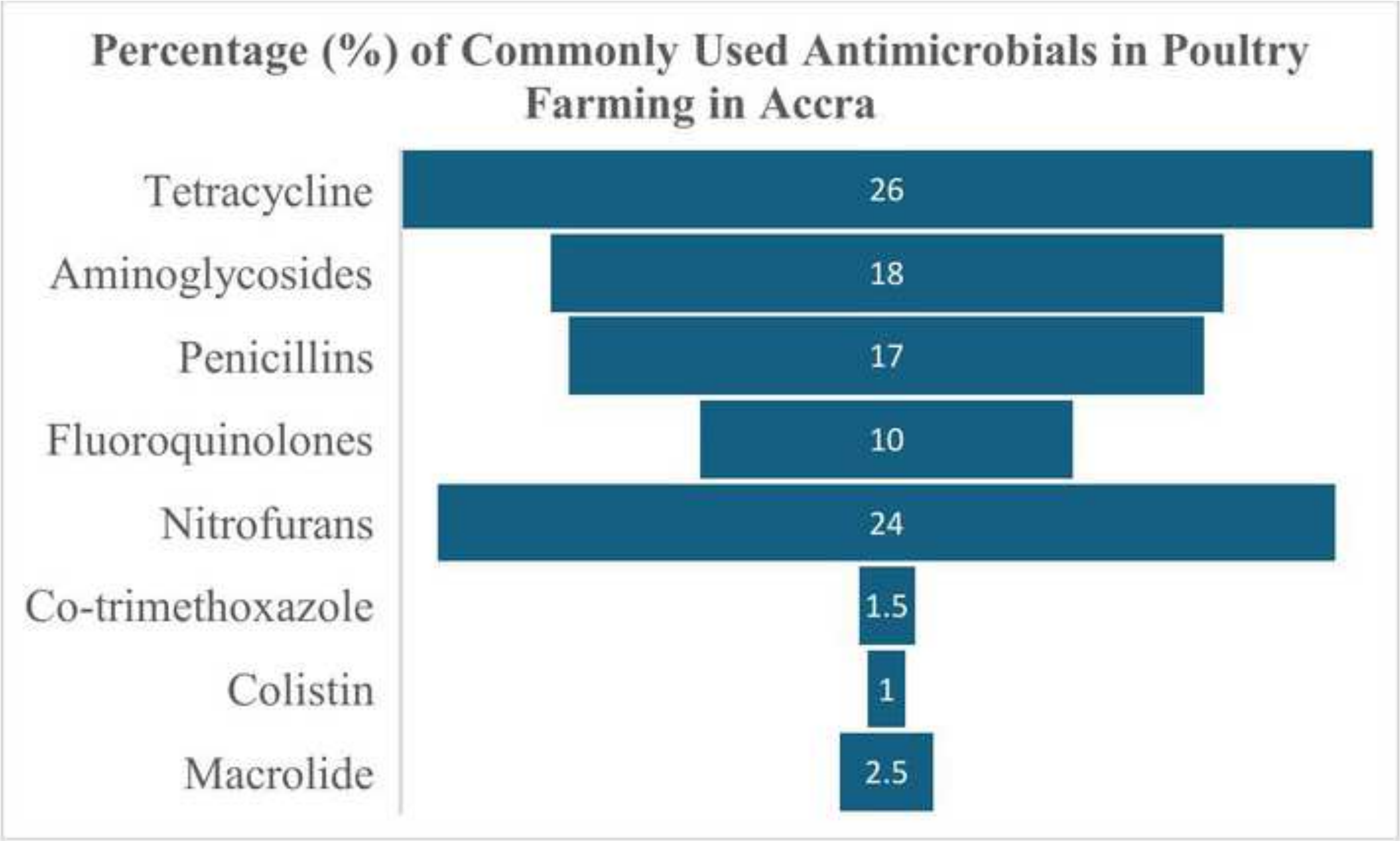
Percentage (%) of Commonly Used Antimicrobials in Poultry Farming in Accra
Tetracycline
26
Aminoglycosides
18
Penicillins
17
Fluoroquinolones
10
Nitrofurans
24
Co-trimethoxazole
1.5
Colistin
1
Macrolide
2.5

Fig 1

Click here to access/download
Supporting Information
S1 File.docx

Click here to access/download
Supporting Information
STROBE-checklist-v4-combined-PlosMedicine.docx
