# Supplementary material for: Awareness of antimicrobial resistance and antibiotic use among poultry farmers in Accra, Ghana: A cross-sectional survey
Source: PLoS One. 2025 Dec 5;20(12):e0337531. doi: 10.1371/journal.pone.0337531 (PMC12680252; doi:10.1371/journal.pone.0337531)
Supplement: Attachment — Submitted filename: Review report by Jean Bosco Ntivuguruzwa.docx [file pone.0337531.s005.docx]

**General comments**

The authors present the results of the original research on the awareness of poultry farmers on the prudent use of antimicrobials. Conclusions are drawn from the obtained data. The study investigated the knowledge, attitudes, and practices of poultry farmers. Inappropriate antimicrobial use is prevalent in the study area, Ghana and the awareness is low among poultry farmers. However, the positive attitudes positive attitudes toward seeking advice and supporting regulations provide a hopeful avenue for change. There is an urgent need for public health interventions and stricter regulatory enforcement to promote the prudent use of antibiotics in food-producing animals. Such measures will protect the efficacy of critical antibiotics for both animal and human medicine. The problem of AMR in agriculture spans across sectors, reinforcing the importance of a One Health approach: coordinated action that involves veterinary services, public health officials, farmers, and environmental agencies.

However, the study has some limitations including the low knowledge of names/families of antibiotics used by poultry farmers, and lack of bacteriological experiments of AMR. However, authors developed strategies to overcome the limitations including using antibiotic’s package images and reporting resistance findings of *Salmonella, E. Coli*, and *Staphylococcus* from other studies.

**Areas of improvement**

Line 55: in the key words section, delete Bacterial species including Escherichia coli, salmonella which were not studies for AMR in this study.

Lines 62, 65 etc. [3][4]. Combine references in one blanket [3-4] and harmonize for the whole manuscript.

Table 2. Replace the question by a positive statement for consistency. Replace “can you reduce AMR development?” by “avoiding overuse of antimicrobials in animal production can reduce AMR development”

Replace “Can your imprudent use of antimicrobials affect the health of others in the form of AMR?” by “Imprudent use of antimicrobials can affect the health people in the form of AMR”

**Line 183.** Delete “When asked *What do you do when your animals get sick?”*

**Authors have included several discussion statements in the results section. Some of the same statements are repeated in the discussion section. Authors should describe their findings in the results section and move the discussion statement to the discussion section. Some examples:**

**Lines 188-190:** These findings show that about 70% of farmers resort to treating their sick poultry without professional veterinary care, either by themselves or with peer advice, which raises concerns about inappropriate drug use,

**Lines 203-206:** This suggests that although veterinary involvement in actual treatment is low, about two-thirds of farmers at least speak with a veterinary or animal drug vendor to get suggestions for what drugs to buy – but one-third engage in completely unguided antibiotic purchases,

**Lines 215 -217:** an injudicious practice where low doses of antibiotics are added to feed to boost weight gain. Although this 4% appears as a small minority, it reflects the persistence of growth promotion uses that have been banned or discouraged in many countries due to AMR concerns.),

**Lines 221-226:** This indicates that while some farmers do access antibiotics through formal veterinary channels (clinics or pharmacies), an even larger share rely on informal local shops. In all cases, antibiotics are readily available without strict prescription enforcement. The ease of access to antimicrobials in the community – often without regulation – was confirmed by many respondents and is consistent with reports from other low-income settings [24]

**Line 237** – the criteria for Positive attitudes should be clearly explained in method section

**Lines 243-245:** This level of agreement --------- should move to discussion section

**Lines 252-253:** The mixed of responses ……………….. should be part of discussion section

**Line 271:** This reflects …. Part of discussion section

**Lines 277 -280:** This suggests that ……………… part of the discussion section

**Lines 283 – 286:** The arguments are mixed and confusing. (53% agreements for education then 70%). Is the idea in line 282 the same like in 283 (specifically ……)? Rephrase to make sense If not better to start a new paragraph to sum up all attitude’s responses.

**Lines 287 – 290:** it is evident ……………. Part of discussion

**Lines 291-298:** Part of discussion section

**Lines 303-308:** part of discussion section

**Lines 313 – 315:** Part of discussion

**Lines 303 -305:** Antibiotics usage – looking at the education of farmers one can wonder their level of knowledge with antibiotics names

**Lines 326 – 339:** part of discussion

**Lines 374 - 389:** part of discussion

**Discussion:** Authors would focus their discussion to the important findings and exclude non-significant predictors of AMR of this study such as education and geography (rural and urban)**.**

**Lines 510 -516:** include references to support your statements

**Lines 532 – 358:** authors present extensive limitations which undermine the quality of the study. They can limit limitations to absent of bacteriological AMR experiments, and knowledge of previously used antibiotics among majority of poultry farmers.
