# Supplementary material for: Awareness of antimicrobial resistance and antibiotic use among poultry farmers in Accra, Ghana: A cross-sectional survey
Source: PLoS One. 2025 Dec 5;20(12):e0337531. doi: 10.1371/journal.pone.0337531 (PMC12680252; doi:10.1371/journal.pone.0337531)
Supplement: Attachment — Submitted filename: Response to Reviewers 2.docx [file pone.0337531.s008.docx]

Accra Technical University

P. O. Box GP 561

Accra – Ghana

05^th^ November 2025

The Academic Editor,

PLoS ONE Journal.

Dear Sir,

**REBUTTAL LETTER:**

**PONE-D-25-35681: AWARENESS OF ANTIMICROBIAL RESISTANCE AND ANTIBIOTIC USE AMONG POULTRY FARMERS IN ACCRA, GHANA: A CROSS-SECTIONAL SURVEY**

I write to express my sincere gratitude for making time off busy schedules to thoroughly review this manuscript and recommend revisions to enhance the quality and clarity of the article. Kindly find below the responses to the minor issues raised by the reviewers and the lines/pages where the corrections or responses could be found as shown in the table below:

| **S/N** | **POINTS RAISED BY REVIEWERS** | **RESPONSE / CORRECTIONS** | **LINES / PAGES IN REVISED MANUSCRIPT** |
| --- | --- | --- | --- |
| 1 | **POINTS RAISED BY REVIEWER 1** |  |  |
|  | The authors have improved the manuscript to bring more clarity. Here are some minor comments:  - Please revise table 5 : For Education the Total of farmers with ASM does not add up to 280 as for other factors.  - It seems like all respondents agreed to be or were identified as Antimicrobial users. to avoid confusion, please add this data somewhere in the manuscript (all respondents were identified as using antibiotics in their animals.  - Line 466: Rectify: antimicrobial misuse causing AMR…. Instead of antimicrobial use causing AMR…. | I am grateful for the thorough review to enhance the quality and clarity of the manuscript.  Table 5 has been revised as suggested  This has been done as advised.  This has been rectified | Line 328 / Page 16  Line 273 / Page 14 |
| 2 | **POINTS RAISED BY REVIEWER 2** |  |  |
|  | All comments were addressed.  However, I have two suggestions:  1) authors added two tables 6, and 7 which are consecutive in the manuscript. Tables 5 and 6 should be placed next to the paragraph they are cited (between 299-300).  2) The discussion section has subheadings which is not common in the discussion section. | Thank you for the relevant recommendations which has enhanced the quality of the manuscript.  This has been done as advised.  This was done in response to an earlier recommendation by one of the reviewers.  Moreso, it conforms to the guidelines on Results, Discussion and Conclusions by the PLOS One journal  “These sections may all be separate or may be combined to create a mixed Results/Discussion section (commonly labeled "Results and Discussion") or a mixed Discussion/Conclusions section (commonly labeled "Discussion"). These sections may be further divided into subsections, each with a concise subheading, as appropriate. These sections have no word limit, but the language should be clear and concise.” | Lines 301-304 / Page 15-16 |

Thank you.

Yours sincerely

Henry Kwadwo Hackman

(Corresponding Author)
